# Distinctive Steady-State Heart Rate and Blood Pressure Responses to Passive Robotic Leg Exercise during Head-Up Tilt: A Pilot Study in Neurological Patients

**DOI:** 10.3389/fphys.2017.00327

**Published:** 2017-06-02

**Authors:** Amirehsan Sarabadani Tafreshi, Robert Riener, Verena Klamroth-Marganska

**Affiliations:** ^1^Sensory-Motor Systems Lab, Department of Health Sciences and Technology, Institute of Robotics and Intelligent Systems, ETH ZurichZurich, Switzerland; ^2^Reharobotics Group, Medical Faculty, Spinal Cord Injury Center, Balgrist University Hospital, University of ZurichZurich, Switzerland

**Keywords:** rehabilitation, robotic tilt table, orthostatic hypotension, stepping speed, exercise intensity, cardiovascular system, linear mixed models, parametric bootstrap

## Abstract

**Introduction:** Robot-assisted tilt table therapy was proposed for early rehabilitation and mobilization of patients after diseases such as stroke. A robot-assisted tilt table with integrated passive robotic leg exercise (PE) mechanism has the potential to prevent orthostatic hypotension usually provoked by verticalization. In a previous study with rather young healthy subjects [average age: 25.1 ± 2.6 years (standard deviation)], we found that PE effect on the cardiovascular system depends on the verticalization angle of the robot-assisted tilt table. In the current study, we investigated in an older population of neurological patients (a) whether they show the same PE effects as younger healthy population on the cardiovascular system at different tilt angles, (b) whether changing the PE frequency (i.e., stepping speed) influences the PE effect on the cardiovascular system, (c) whether PE could prevent orthostatic hypotension, and finally, (d) whether PE effect is consistent from day to day.

**Methods:** Heart rate (HR), and systolic and diastolic blood pressures (sBP, dBP) in response to PE at two different tilt angles (α = 20°, 60°) with three different PE frequencies (i.e., 0, 24, and 48 steps per minute) of 10 neurological patients [average age: 68.4 ± 13.5 years (standard deviation)] were measured on 2 consecutive days. Linear mixed models were used to develop statistical models and analyze the repeated measurements.

**Results:** The models show that: PE significantly increased sBP and dBP but had no significant effect on HR. (a) Similar to healthy subjects the effect of PE on sBP was dependent on the tilt angle with higher tilt angles resulting in a higher increase. Head-up tilting alone significantly increased HR and dBP but resulted in a non-significant drop in sBP. PE, in general, had a more additive effect on increasing BP. (b) The effect of PE was not influenced by its speed. (c) Neither during head-up tilt alone nor in combination with PE did participants experience orthostatic hypotension. (d) The measurement day was not a statistically significant factor regarding the effects of verticalization and PE on the cardiovascular response.

**Conclusion:** We provide evidence that PE can increase steady-state values of sBP and dBP in neurological patients during head-up tilt. Similar to healthy subjects the effect on sBP depends on the verticalization angle of the robot-assisted tilt table. PE might have the potential to prevent orthostatic hypotension, but as the amount of drop in BP in response to head-up tilting was not leading to orthostatic hypotension in our patients, we could neither conclude nor reject such a preventive compensatory effect. Furthermore, we found that changing the PE speed does not influence the steady-state cardiovascular response.

## 1. Introduction

**Figure 1:**
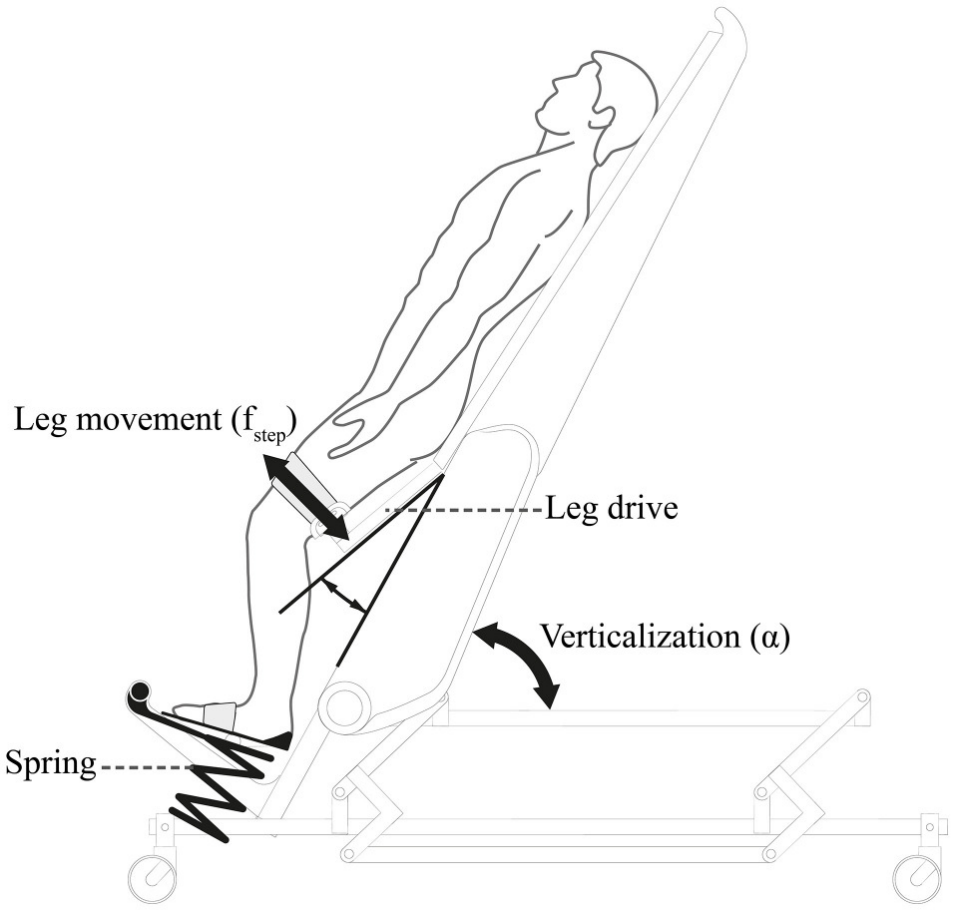
**The robot-assisted tilt table Erigo®: Erigo® combines verticalization and cyclic passive robotic leg movements [i.e., leg exercise (PE)] to enhance blood circulation and potentially to prevent orthostatic hypotension**. Picture is copyrighted by Hocoma AG Switzerland and is adapted with permission.

Critically ill patients (e.g., after neurological diseases such as stroke or spinal cord injury, SCI) often have to stay in bed for days and weeks. This prolonged bed rest might lead to secondary complications that delay or even prevent patients' recovery (Dittmer and Teasell, 1993; Teasell and Dittmer, 1993; Brower, 2009). Mobilization can ameliorate such adverse side effects and improve functional recovery (Morris, 2007; Burtin et al., 2009; Bourdin et al., 2010). However, cardiovascular autonomic dysfunction (Ravensbergen et al., 2013; West et al., 2015) and in particular, orthostatic hypotension is prevalent among these patients (Illman et al., 2000; Feldstein and Weder, 2012; Ravensbergen et al., 2013) and is a barrier for mobilization. Head-up tilt was originally applied to investigate orthostatic hypotension as part of examining the autonomic nervous system function (Pagani et al., 1986). For such diagnostic purposes, usually mechanical head-up tilt tables were used. However, in recent years, head-up tilt tables have also been used for rehabilitation. As a potential solution for safe mobilization, a robot-assisted tilt table has been suggested (Colombo et al., 2005). Feasibility in early rehabilitation of patients with neurological disorders was demonstrated (Craven et al., 2013; Kuznetsov et al., 2013; Frazzitta et al., 2015). In contrast to conventional tilt tables, robot-assisted tilt tables (Erigo, Hocoma AG, Switzerland) have an integrated robotic module, which allows passive robotic leg exercise (PE) training (Figure 1). The PE mechanism has been utilized for developing biofeedback systems (Giggins et al., 2013) for early rehabilitation, either without contribution (Wieser et al., 2014; Sarabadani Tafreshi et al., 2015, 2017a) or with partial active participation (Laubacher et al., 2015; Saengsuwan et al., 2015a,b) of the patient in the loop, in order to drive cardiovascular variables or performance to desired levels. However, the main objective for PE in the robot-assisted tilt table is the ability to enhance blood circulation and thus, avoid orthostatic hypotension (Czell et al., 2004; Colombo et al., 2005). Mobilization through head-up tilting alone might result in premature termination of the therapy due to orthostatic reactions leading to adverse events such as dizziness or presyncope, whereas involving PE might prevent such conditions and enable an effective therapy without termination (Kuznetsov et al., 2013). Moreover, training together with PE might be more efficient in improving leg strength and cerebral blood flow than head-up tilting alone without PE (Kuznetsov et al., 2013).

By definition, orthostatic hypotension is a drop of more than 20 mmHg (30 mmHg in patients with supine hypertension) in systolic blood pressure (sBP) or more than 10 mmHg in diastolic blood pressure (dBP) (Freeman et al., 2011). The application of robot-assisted tilt tables in rehabilitation increases orthostatic tolerance and reduces the occurrence of orthostatic hypotension over time (Taveggia et al., 2015). In addition to long-term effectiveness, training with a robot-assisted tilt table is also effective in the short-term prevention of orthostatic hypotension and resultant syncope. Comparison of the robot-assisted tilt table with regular tilt tables has shown that it results in a lower number of syncopes in both healthy subjects and patients (Czell et al., 2004; Luther et al., 2008; Kuznetsov et al., 2013). It is known that PE evokes a cardiovascular response in healthy subjects (Chi et al., 2008) and patients (Yoshida et al., 2013). However, most of these studies have been limited in investigating the effect of PE at an arbitrarily chosen single intensity (i.e., PE frequency) and one single tilt angle in the range of α = 60°– 75°. Although, some preliminary explorations and assumptions have been done about the effect of performing PE at different frequencies or different tilt angles (Wieser, 2011; Wieser et al., 2014), a systematic study is missing. In our previous work conducted with a rather young healthy population [average age: 25.1 ± 2.6 years (standard deviation)], we found that PE influences the cardiovascular system's response systematically. However, this effect is highly dependent on the angle of tilt at which PE is performed; the PE effect is not present at each angle and not always in the same direction (i.e., increase or decrease of cardiovascular variables). Thus, we concluded that the potential preventive effect of PE on orthostatic hypotension is dependent on the verticalization angle of the robot-assisted tilt table (Sarabadani Tafreshi et al., 2016). Aging (Jones et al., 2003) and neurologic diseases (Benarroch, 2008) can lead to a depressed baroreflex sensitivity. In the current work, we aimed at exploring the PE effect in an older population of neurological patients, where a depressed baroreflex sensitivity and thus, a stronger cardiovascular response to the PE or head-up tilting was expected. We hypothesized that; (a) similar to young healthy subjects, in neurological patients the PE effect on the cardiovascular system is tilt-angle dependent; (b) the PE frequency plays a major role in the PE effect on the cardiovascular system's response, and a higher PE frequency systematically results in a higher effectiveness; (c) the PE has a compensatory effect which can prevent orthostatic hypotension when dealing with orthostatic challenge; (d) the PE effect is systematically replicable and consistent from day to day. To examine our hypotheses, we measured heart rate (HR), sBP, and dBP of ten neurological patients [average age: 68.4 ± 13.5 years (standard deviation)] in response to PE at three different frequencies [i.e., 0, 24, and 48 steps per minute (min)] and at two different tilt angles (i.e., α = 20°, 60°), on 2 consecutive days.

It is worth mentioning that in our previous study (Sarabadani Tafreshi et al., 2016), we had also tested the effect of the simultaneous application of functional electrical stimulation (FES) to leg muscles during the PE on HR, sBP, and dBP. Since our investigation (Sarabadani Tafreshi et al., 2016) and furthermore, other patient studies investigating deployment of FES together with PE (Chi et al., 2008; Craven et al., 2013; Kuznetsov et al., 2013; Yoshida et al., 2013) had found no significant contribution from the FES to the PE effect, in the current study, we omitted investigation of the FES effect. Instead, we investigated whether changing the PE frequency (i.e., stepping speed) influences the PE effect [see Hypothesis (b) above].

## 2. Materials and methods

### 2.1. Robot-assisted tilt table

Erigo® (Hocoma AG, Volketswil, Switzerland) is a robot-assisted tilt table designed for rehabilitation of patients facing bed rest (Colombo et al., 2005). The table is enhanced with a motor-driven stepping device that can provide PE through two leg drives (Figure 1). The stepping frequency *f*_*step*_ can be continuously adjusted between 0 and 80 steps per min with equal periods of extension and flexion phases. Simultaneous to the robotic leg movement, cycling leg loading is provided through two springs located beneath the patient's feet. The tilt table verticalization angle α can be continuously adjusted between 0 and 75°.

### 2.2. Measurement equipment

The raw blood pressure (BP) signal was measured non-invasively using a CNAP® monitor 500 (CNSystems Medizintechnik AG, Austria). The monitor uses a double-finger and an arm cuff to measure the BP signal. Before each measurement, about 2 min for calibration are required. To record the data in real-time, the BP signal from the monitor (100 Hz) was fed into a g.USBamp biosignal amplifier (g.tec medical engineering GmbH, Austria) connected to a laptop through a standard USB connection. The data was recorded using a MATLAB®/Simulink model (Mathworks Inc., Natick, MA, United States) through the provided API for the biosignal amplifier (g.tec medical engineering GmbH, Austria).

### 2.3. Subjects

Neurological patients were recruited from Zürcher Höhenklinik Wald, a neurorehabilitation center in the metropolitan area of Zurich. The study was approved by Kantonale Ethikkommission Zürich and Swissmedic, and it was registered at ClinicalTrials.gov (registration identifier NCT02268266). Criteria for participation included post-acute phase after stroke or other neurological functional disorder, oxygen saturation of the blood of at least 92% (measured by pulse oximetry), resting HR 40–100 beats per min (bpm), resting sBP 120–220 mmHg, no severe contraction in the legs (modified Ashworth scale >3). For more details, see clincialtrials.gov (identifier NCT02268266).

### 2.4. Experimental protocol

**Figure 2:**
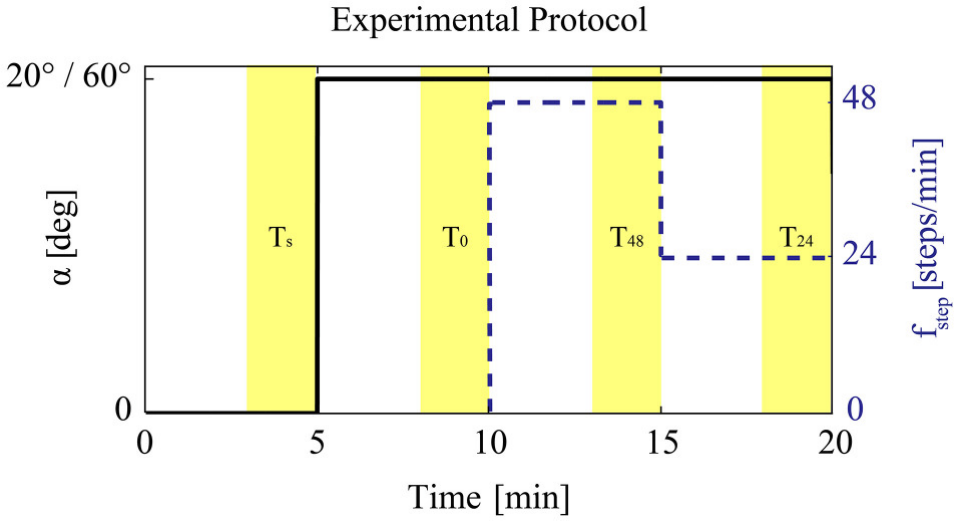
**Experimental protocol: After 5 min in the supine position, the subject was either tilted to α = 20° or α = 60° in a random order**. During the head-up tilt, PE with 0, 24, and 48 steps per min was performed. The order of performing various leg exercise frequencies at each angle was randomized.

Each patient was measured at two different head-up tilt angles (α = 20° and α = 60°) and three different PE frequencies (0, 24, and 48 steps per min) in a random order (Figure 2). Temporary cardiovascular changes such as overshoots could occur due to the sudden changes in the applied external stimuli (e.g., sudden change of PE frequency from 0 to 24 steps per min) (Sarabadani Tafreshi et al., 2017b). To avoid considering transient reactions in our evaluations, we focused on the steady-state responses. Transient responses to head-up tilting alone settle down within the first 30 s after tilting (Toska and Walløe, 2002). Moreover, based on our previous studies we considered that the time required to reach the steady-state responses to changes in PE frequency is < 5 min (Wieser et al., 2014). Therefore, each combination of angle and PE frequency was measured for 5 min. Measurement at each angle (three PE frequencies at α = 20° and α = 60°, 15 min for each angle) was preceded by a 5 min measurement period in the supine position. Furthermore, before starting each measurement, an initial 5 min period for a short break and recalibration of the BP monitor (calibration break) was considered. Accordingly, the total measurement time in each experimental session was 40 min per patient (Figure 2). The BP monitor was recalibrated before starting the measurement for each angle.

The measurements were performed on 2 consecutive days. For the experimental session on the first day, the order of presenting the angles and PE frequencies for each patient was randomized. The second-day experiment was a replication of the one of the first day.

Before each experimental session, the patient was positioned on the robot-assisted tilt table by the therapist, and the table was adjusted to patient's height. The legs were placed in the cuffs and adjusted to assure foot loading. The range of motion for hip and knee joints of each leg was measured using the table sensors and provided to the table software to ensure robotic leg movements within these borders. For BP measurement, the arm and finger cuff of the BP monitor were mounted on one arm. An armrest module was used to fix and keep the arm at heart level during measurements.

### 2.5. Signal processing and statistical analysis

The raw BP signal in the last 2 min of each experimental condition (i.e., supine, PE at 0, 24, and 48 steps per min) was analyzed to compute steady-state values of HR, sBP, and dBP (Wieser et al., 2014; Sarabadani Tafreshi et al., 2016). To compute real-time values of sBP (maxima of BP signal) and dBP (minima of BP signal), the BP signal peaks were identified using a peak detection algorithm in MATLAB® (Mathworks Inc., Natick, MA, United States) and then were visually inspected to ensure correctness and completeness of the detected peaks. Consecutive dBP peaks were used to compute heart period and accordingly real-time HR signal. The calculated HR, sBP, and dBP biosignals during the last 2 min of each experimental condition (i.e., *T*_*s*_, *T*_0_, *T*_24_, *T*_48_ in Figure 2, corresponding to last 2-min periods of supine, and PE at 0, 24, and 48 steps per min) were averaged to calculate the steady-state values in each condition (i.e., μ_*s*_, μ_0_, μ_24_, μ_48_). In periodic breathing (a phenomenon seen in chronic heart failure patients), the BP oscillations (0.02–0.4 Hz; Parati et al., 1995) could be overlaid by a very low frequency (VLF) rhythmic pattern (around 0.02 Hz) (Mortara et al., 1997; Francis et al., 2000; Pinna et al., 2000). In such cases, the considered 2 min periods to calculate the steady-state values, do not necessarily include an integer number of VLF cycles. Thus, simple averaging does not cancel the VLF effect, and the calculated steady-state values might not completely represent the changes emerging from PE. Furthermore, our measurements and thus, the considered 2 min periods do not necessarily always start at the same phase of the VLF cycle such that when we, for example, compute the differences between the steady-state values, the VLF effect automatically gets canceled. Therefore, before calculation of steady-state values for these patients, we deployed a fast Fourier transform (FFT) algorithm to find the corresponding frequency of these oscillations related to periodic breathing and then applied moving average windowing with a window size equal to the period of the VLF oscillation. After compensation of the effect of VLF oscillation in the last 2 min of each condition, we averaged the signals to calculate the steady-state values.

### 2.6. Statistical procedure

To analyze the repeated measurements and to perform Linear mixed model analyses (Bates et al., 2014) we used R-package (R Core Team, 2015; version 3.2.0). In contrast to repeated measure ANOVA which requires balanced data, linear mixed models can deal with unbalanced data (Verbeke and Molenberghs, 2009). Therefore, this method enabled us to analyze the recorded data from all patients without omitting any data points. The statistical procedure consisted of four steps (Sarabadani Tafreshi et al., 2016):

1. **Model assessments:** We considered an initial hypothesized model and applied maximum likelihood (ML) algorithm to fit models with a various mixture of fixed and interaction terms (considered in the initial model) to the data. Then we used Akaike information criterion (AIC) and Bayesian information criterion (BIC) scores to compare these models and recognize which main and interaction factors are important to describe the data; those terms which were not significantly improving the fit were removed from the initial model to achieve a final model describing the behavior of each cardiovascular variable with respect to the considered terms.
2. **Calculation of *p*-values:** Since computation of denominator degrees of freedom for the test statistic in the case of linear mixed models is not straightforward, calculation of *p*-values for these models is a controversy (Bates et al., 2014). However, parametric bootstrap has been suggested as a reliable approach for constructing *p*-values (Bates et al., 2014). Therefore, having found a model with relevant terms, we constructed *p*-values for each relevant term by performing parametric bootstrap (Bates et al., 2014) with 10,000 samples while fitting the models using the ML algorithm. For this purpose, we eliminated each relevant term once at a time and using the likelihood ratio test (LRT) and supposing that it has a chi-square distribution, we compared the reduced and full models. Following this, we obtained a chi-square value (observed LRT statistic) from each comparison. Afterward, we randomly generated new data points under the fitted reduced model with one term less (i.e., null hypothesis). We then used the ML algorithm to refit the reduced and full models on this new simulated data [parametric bootstrap (Bates et al., 2014)] and calculated a simulated LRT statistic value by comparing the two models. This scheme was performed 10,000 times resulting in 10,000 simulated LRT statistic values under the null hypothesis. To calculate the corresponding *p*-value for the relevant term under consideration, we then computed the proportion of the simulated LRT statistic values that are bigger or equivalent to the observed LRT statistic value (Halekoh and Højsgaard, 2014). However, this *p*-value calculation approach could not be used when the final model (the result of Step 1) consisted of only an intercept term (the average of data, i.e., the simplest possible model). For such cases, we tested whether the intercept is statistically significantly different from zero. By definition *p*-value for double tailed events can be computed by 2 × *MIN*{*Pr*(*T* ≤ *t*_*obs*_; *H*_0_), *Pr*(*T* ≥ *t*_*obs*_; *H*_0_)} where *Pr* stands for probability, *H*_0_ for null hypothesis, *T* is a continuous random variable, and *t*_*obs*_ is an observed value (here, *t*_*obs*_ = 0) (Cox and Hinkley, 1979). Therefore, to calculate the *p*-value examining the significance of the intercept of intercept-only models from zero, we first bootstrapped coefficients for the intercept and computed the fractions of coefficient values higher (*p*^+^) and smaller (*p*^−^) than zero. We then calculated the *p*-value by multiplying the minimum value of the two fractions by two [i.e., 2 × *MIN*(*p*^+^, *p*^−^)].
3. **Final model:** Following the calculation of the *p*-values, we used the Restricted Maximum Likelihood (REML) algorithm to refit the model to obtain the model to be reported. To assure the validity of the model we then checked the normality of the residuals by inspecting the corresponding Q-Q plot and performing a Shapiro-Wilk test (*p* < 0.05 significant violation). The outcome of this step was the reported model parameter estimates and their standard errors (*SE*).
4. **Confidence intervals:** In the last step, we performed another parametric bootstrap with 10,000 samples on the final model (Step 3) to calculate the confidence intervals (CIs) for each model parameter (Bates et al., 2014).

To evaluate the effect of PE, linear mixed model analyses with the dependent variables ΔHR, ΔsBP, and ΔdBP (i.e., changes in HR, sBP, and dBP, respectively) were performed in two phases.

### 2.7. Primary analysis

In the first phase, we focused only on the contribution of PE to the change of cardiovascular variables independent of the head-up tilting effect. To this end, we decoupled the changes introduced by PE from changes introduced by head-up tilting alone. Therefore, we normalized the steady-state values of HR, sBP, and dBP in response to 24 (μ_24_) and 48 (μ_48_) steps per min with respect to 0 (μ_0_) steps per min. This normalization allowed removing the changes introduced by head-up tilting alone from the total changes, and thus, obtaining only changes introduced by PE. Accordingly, the so-called gains of the system, i.e., steady-state changes introduced by only applying the PE at different head-up tilt angles were computed:

(1) $$\Delta Value_{24/48-0}=\mu_{24/48}-\mu_{0}$$

where Δ*Value* corresponds to the relative change in the cardiovascular variable (in bpm or mmHg) by performing PE at a specific tilt angle. Clearly, the reference value (μ_0_) for each head-up tilt angle experiment was different.

To perform linear mixed model analyses, as fixed effects, the potential effect of tilt angle (α), PE frequency (*f*_step_), and measurement day were considered into the initial model. The day was treated as a categorical variable referring to measurement day (1 or 2, i.e., first or second day). To have meaningful intercepts in the models that can be interpreted easier, we shifted the angle term with respect to its value at α = 20° and PE frequency with respect to *f*_step_ = 24 steps per min. Therefore, for the statistical analysis the following initial model was considered:

(2) $$\begin{array}{l} \begin{array}{c} \Delta Value=\beta_{0}+\beta_{1}(f_{\text{step}}-24)+\beta_{2}(\alpha {}^{\circ}-20{}^{\circ}) \end{array}\\ \;\begin{array}{c} +\beta_{3}(f_{\text{step}}-24)\times (\alpha {}^{\circ}-20{}^{\circ})+\beta_{4}\times Day \end{array} \end{array}$$

where Δ*Value* corresponds to the relative change in the cardiovascular variable (in bpm or mmHg) calculated according to the tilt angle and PE frequency based on equation 1 and β_*i*_s are model coefficients to be estimated. As random effects, we considered two terms; one random intercept for each patient, i.e., (1|patient) to account for potential correlation due to repeated measures of the same patients in various conditions as well as to account for sampling the study population from the general population. Furthermore, one random intercept for each experimental day for each patient (1|patient:day) to account for potential random changes induced in the condition of each experiment by changing the measurement day, such as time of day, meals, etc.

### 2.8. Secondary analysis

In the second phase, we also considered the head-up tilting effect in the analysis to evaluate whether PE in comparison to head-up tilting alone has a significant contribution to the changes in cardiovascular variables and furthermore, whether these changes are in opposite or same directions (compensatory vs. additive). To this end, in contrast to primary analysis where we normalized the steady-state values to 0 steps per min (μ_0_), we normalized the steady-state values of HR, sBP, and dBP in response to 0 (μ_0_), 24 (μ_24_), and 48 (μ_48_) steps per min with respect to the supine position steady-state value (μ_*s*_). Accordingly, we had:

(3) $$\begin{array}{c} \Delta Value_{0/24/48-s}=\mu_{0/24/48}-\mu_{s} \end{array}$$

where Δ*Value* corresponds to the relative change in the cardiovascular variable (in bpm or mmHg) with respect to supine position. Clearly, the baseline value (μ_*s*_) for each head-up tilt angle experiment was different.

The outcome of primary analysis showed that PE frequency did not have a significant effect on the change of steady-state cardiovascular variables, i.e., there was no significant difference between performing PE at 24 and 48 steps per min (see below, Section 3.2). Therefore, in the secondary analysis, we did not consider the effect of PE frequency but rather considered PE at both 24 and 48 steps per min, as an occasion of PE. Accordingly, to perform linear mixed model analyses, we considered a categorical variable called PE representing the status of performing PE (equal to 1 for 24 or 48 steps per min, and 0 for 0 steps per min), potential effect of tilt angle (α), and measurement day (treated as categorical variable similar to the primary analysis) as fixed effects into the initial model. To obtain easily interpretable intercepts, the angle term was shifted with respect to its value at α = 20°. Thus, the following initial model was considered for the secondary analysis:

(4) $$\begin{array}{l} \begin{array}{c} \Delta Value=\beta_{0}+\beta_{1}PE+\beta_{2}(\alpha {}^{\circ}-20{}^{\circ}) \end{array}\\ \;\begin{array}{c} +\beta_{3}PE\times (\alpha {}^{\circ}-20{}^{\circ})+\beta_{4}\times Day \end{array} \end{array}$$

with Δ*Value* to be calculated based on equation 3 and β_*i*_s model coefficients to be estimated. As random effects, similar to the primary analysis we considered two terms; one random intercept for each patient, i.e., (1|patient) and one random intercept for each experimental day for each patient (1|patient:day).

## 3. Results

### 3.1. Subjects

Eleven patients were included in the study. One person with diabetes mellitus was mistakenly included although this was an exclusion criterion. The subjects provided written informed consents before the experiments. From the recruited neurological patients, one subject withdrew his consent after being put on the robot-assisted tilt table. Accordingly, 10 patients participated in the study (nine males and one female; see Table 1 for detailed characteristics and demographics). One subject (P6) participated on the first measurement day only. The outcomes of α = 20° experiment of P6 on day 1 (the only day he had participated) and P7 on day 2 were omitted from analysis due to data collection error. In three subjects (P5, P7, and P10) we compensated observed VLF due to periodic breathing (see above, Section 2.5). We observed no adverse events (e.g., dizziness, presyncope, etc.) related to the orthostatic challenge during the experiments.

**Table 1:** **Patients' data**.

**Patient**	**Sex**	**Age (year)**	**Weight (kg)**	**Height (cm)**	**BMI (kg/m^2^)**	**Disease**	**Hemiparetic side**	**Comorbidities**	**Antihypertensive medications**
P1	Male	47	83	178	26.2	CVA	Right	Dyslipidemia, Hypertension	Beta blocker
P2	Male	73	79.8	165	29.3	CIP	Bilateral	Hypertension	ACE Inhibitor
P3	Male	76	74.8	174	24.7	CVA	Right	Dyslipidemia, Hypertension	Ca Antagonist
P4	Male	52	72.1	173	24.1	HIE	Bilateral	Cardiomyopathy	Beta blocker+ACE inhibitor
P5	Male	67	97.2	185	28.4	CVA	Left	Hypertension	Beta blocker+ACE inhibitor
P6	Male	86	70.2	173	23.5	CIP	Bilateral	Hypertension	Beta blocker+ACE inhibitor+Ca antagonist
P7	Male	74	81.6	179	25.5	CVA	Right	Hypertension, Diabetes mellitus	ACE inhibitor
P8	Female	76	74.5	165	27.4	CVA	Right	Hypertension	Beta blocker
P9	Male	81	95.4	178	30.1	CVA	Left	Hypertension	Beta blocker+ACE inhibitor+Ca antagonist
P10	Male	52	77.2	187	22.1	CIP	Bilateral	Hypertension	Beta blocker+ACE inhibitor
Mean		68.4	80.6	175.7	26.1				
Standard deviation		13.5	9.2	7.3	2.6				

*BMI, Body mass index; CVA, Cerebrovascular accident; CIP, Critical Illness Polyneuropathy; HIE, Hypoxic Ischemic Encephalopath*.

### 3.2. Primary analysis

In the primary analysis, we investigated the effect of PE alone (i.e., independent of the head-up tilting effect). PE had significant contributions to sBP and dBP, but not to HR (see Table 2).

**Table 2:** **Statistical models for ΔHR, ΔsBP, and ΔdBP in primary analysis, i.e., consideration of PE effect independent from head-up tilting effect contribution**.

**Cardiovascular variable**	**Parameter**	**Coeff**.	**Estimate**	***SE***	***t*-value**	**95% CI**	***P*-value**
Δ*HR*	*Intercept*	β_0_	0.06	0.36	0.16	-0.65, 0.76	0.870
Δ*sBP*	*Intercept*	β_0_	3.85^*^	1.43	2.69	1.03, 6.67	0.016
	(α°−20°)	β_2_	0.12^***^	0.03	4.09	0.06, 0.18	<0.001
Δ*dBP*	*Intercept*	β_0_	2.42^**^	0.93	2.61	0.61, 4.22	0.006

SE, Standard error; CI, confidence interval. p-values are computed based on the parametric bootstrap and are unadjusted. The signs ^*^, ^**^, and ^***^ show significant findings with p ≤ 0.05, 0.01, and 0.001, respectively. Coeff. parameters represent the coefficients in Equation (2).

**Figure 3:**
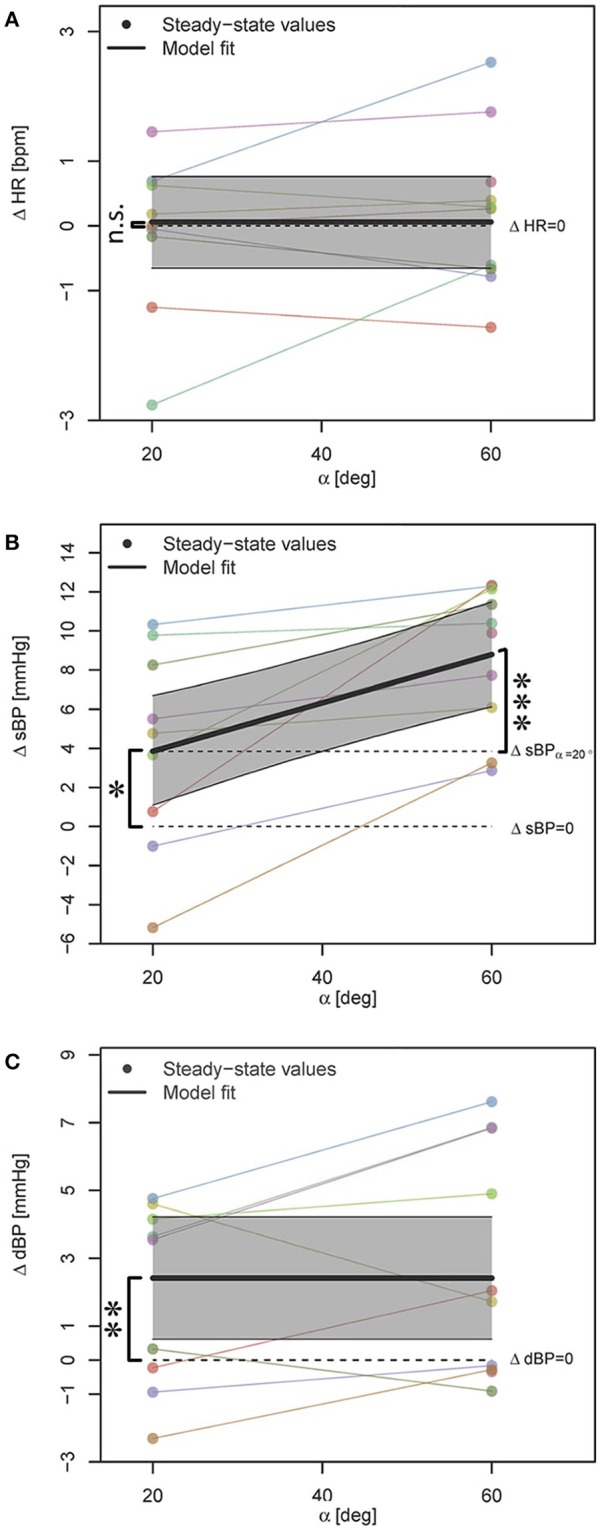
**Statistical models for ΔHR, ΔsBP, and ΔdBP responses to PE (A–C)** when the effect is decoupled from the head-up tilting effect. Data points correspond to the average steady-state response to 24 and 48 steps per min on 2 days. In case of availability of only single case measurement, data point without averaging is shown. The steady-state values for each subject are color-coded and connected with a line. The highlighted areas show 95% CI. The signs ^*^, ^**^, and ^***^ mark significant findings with *p* ≤ 0.05, 0.01, and 0.001, respectively. *n*.*s*. marks non-significant differences.

Hypothesis (a): PE at α = 20° resulted in significant increase of sBP by about 4 mmHg (see Table 2, β_0_, see also Figure 3B). This influence at α = 60° was pronounced and the amount of increase was significantly higher (about 4.8 mmHg) as compared to α = 20°, confirming the tilt-angle dependency of the PE influence on sBP (see Table 2, β_1_, see also Figure 3B). For dBP, although PE in general resulted in significant increase (about 2.5 mmHg, see Table 2, β_0_, see also Figure 3C), no tilt-angle dependency of the PE influence could be concluded. For HR, no significant contribution was observed (see Table 2, β_0_, see also Figure 3A).

Hypothesis (b): Increasing the PE frequency did not result in any significant contribution to the PE effect on any of the cardiovascular variables (neither increase nor reduction of the PE influence). However, when comparing initial models for ΔsBP (see above, Step 1 of the statistical procedure in Section 2.6), we found two best models where AIC and BIC scores did not agree. Although, both models provided evidence for tilt-angle dependency of PE influence on sBP (β_2_ coefficient in Equation 2), one model also showed a borderline significant trend (parametric bootstrap *p* = 0.064) toward contribution from PE frequency (β_1_ coefficient in Equation 2) with rather a small effect size (β_1_ ≃ 0.09, i.e., ΔsBP = 2.2 mmHg). As the influence of this parameter was rather small, we chose the model with the better (lower) BIC score (i.e., simpler model), only considering the tilt angle influence, to avoid potential overfitting.

Hypothesis (d): Measurement day did not have a significant effect on any variable.

### 3.3. Secondary analysis

**Figure 4:**
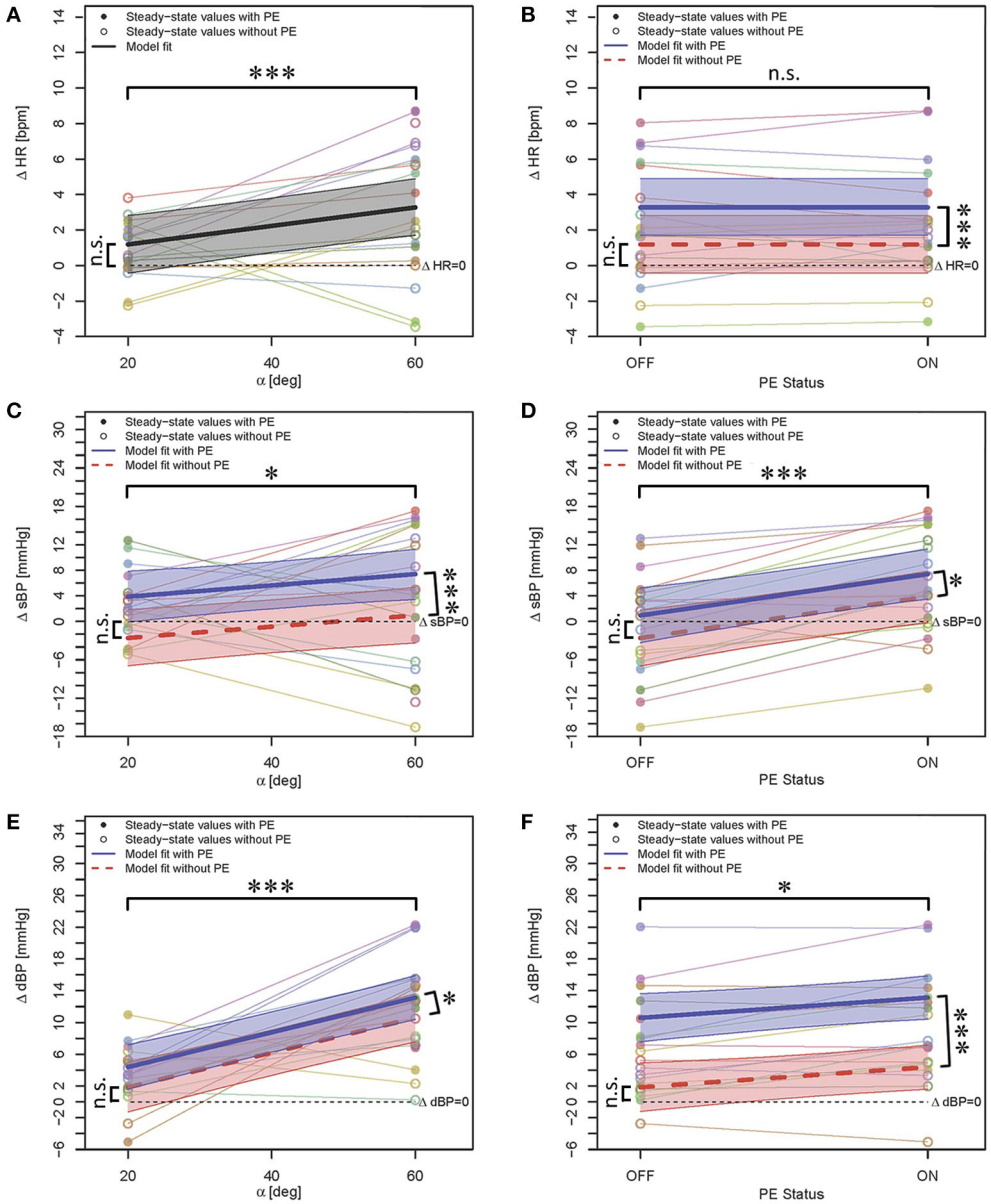
**Statistical models for ΔHR (A,B)**, ΔsBP **(C,D)**, and ΔdBP **(E,F)** responses to PE when the effect is considered together with the effect of head-up tilting. In this figure, both columns show the same models with variation with respect to tilt angle (left column) and PE status (right column), respectively. As the PE effect for ΔHR was not significant, Figure 4A, only shows one model for both conditions of with and without PE. Data points correspond to the average of steady-state response on two days. In case of availability of only single case measurement, the data point without averaging is shown. The steady-state values for each subject in each case (PE status or tilt angle) are color-coded and connected with lines. The highlighted areas show 95% CI. The signs ^*^ and ^***^ mark significant findings with *p* ≤ 0.05 and 0.001, respectively. *n*.*s*. marks non-significant differences.

In the secondary analysis we investigated the effect of PE combined with head-up tilting effect. ΔHR at α = 20° was not influenced significantly by head-up tilting or by PE (see Table 3, β_0_, see also Figures 4A,B). We found a significant increase in HR when tilted to α = 60° in comparison to α = 20° (about 2 bpm, see Table 3, β_2_, see also Figures 4A,B) but PE did not have a significant contribution to the changes (see Table 3, see Figures 4A,B). This was expected as in the primary analysis no systematic contribution of PE on ΔHR was observed (see Figure 3A).

**Table 3:** **Statistical models for ΔHR, ΔsBP, and ΔdBP in secondary analysis, i.e., when PE and head-up tilting effects are considered together**.

**Cardiovascular variable**	**Parameter**	**Coeff**.	**Estimate**	***SE***	***t*-value**	**95% CI**	***P*-value**
ΔHR	*Intercept*	β_0_	1.19	0.82	1.45	-0.41, 2.81	0.173
	(α°−20°)	β_2_	0.05^***^	0.01	4.50	0.03, 0.08	<0.001
ΔsBP	*Intercept*	β_0_	−2.58	2.24	–1.15	–6.95, 1.82	0.239
	PE	β_1_	6.45^***^	1.56	4.13	3.32, 9.46	<0.001
	(α°−20°)	β_2_	0.09^*^	0.04	2.35	0.01, 0.16	0.023
ΔdBP	*Intercept*	β_0_	1.84	1.58	1.17	-1.27, 4.86	0.236
	PE	β_1_	2.55^*^	1.19	2.14	0.25, 4.86	0.036
	(α°−20°)	β_2_	0.22^***^	0.03	7.64	0.16, 0.27	<0.001

SE, Standard error; CI, confidence interval. p-values are computed based on the parametric bootstrap and are unadjusted. The signs ^*^ and ^***^ show significant findings with p ≤ 0.05 and 0.001, respectively. Coeff. parameters represent the coefficients in Equation (4).

Hypothesis (c): Head-up tilting alone (without PE) to α = 20° had no statistically significant effect on sBP (see Table 3, β_0_, see also Figures 4C,D). We observed a trend toward sBP drop at α = 20° (without PE) as compared to the supine position. No drop at α = 60° (without PE) was observed (see Figures 4C,D). ΔsBP increased significantly with PE (about 6.45 mmHg, see Table 3, β_1_, see also Figures 4C,D) and with change in tilt angle from α = 20° to α = 60° (about 3.6 mmHg, see Table 3, β_2_, see also Figures 4C,D). These results show that PE had a very slight compensatory (only at α = 20°) but more additive effect on increasing sBP (see Figures 4C,D) in our study population.

The effect of head-up tilting alone (without PE) to α = 20° on dBP was not statistically significant although a positive trend could be observed (see Table 3, β_0_, see also Figures 4E,F). PE and change in head-up tilt angle from α = 20° to α = 60° both resulted in statistically significant increase of dBP (see Table 3, β_1_ and β_2_, respectively, see also Figures 4E,F) with about 2.5 and 8.8 mmHg increase, respectively. This clearly shows the additive effect of PE on the head-up tilting effect on increasing dBP.

Hypothesis (d): Measurement day did not have a significant effect in any case.

## 4. Discussion

We observed that PE alone increased sBP and dBP in a statistically significant manner but had no significant effect on HR during head-up tilt in neurological patients. Only for sBP, the effect was dependent on the tilt angle, i.e., performing PE at higher angles resulted in higher increase in sBP.

In our previous study in a healthy population (Sarabadani Tafreshi et al., 2016), we had observed similar but to some extent different results: PE during head-up tilt had resulted in significant changes in HR, sBP, and dBP. However, changes in both, sBP and dBP were dependent on the tilt angle. Furthermore, these changes were not always in the same direction (i.e., increase or decrease of the cardiovascular variables) (Sarabadani Tafreshi et al., 2016). In the current study, the contribution of PE on sBP and dBP (independent from the tilt angle) was always positive and in the same direction, resulting in significant increases of sBP and dBP. This observation might explain the protective effect of PE on orthostatic hypotension: significantly fewer people (both, healthy subjects and patients) experience orthostatic hypotension on a robot-assisted tilt table where PE is included (as compared to a standard tilt table) at a tilt angle between α = 60°−75° (Czell et al., 2004; Luther et al., 2008; Kuznetsov et al., 2013).

Although the majority of patients took cardioactive medication (e.g., beta blockers in 7 out of 10 subjects, see Table 1), the amount of change in BP was relatively higher to that of healthy subjects. However, in contrast to healthy subjects, in the patients, we did not observe any significant changes in HR in response to PE. We only observed a slight increase of HR in response to head-up tilting (about 3.2 bpm see Table 3, ΔHR model). In our former study on healthy subjects (Sarabadani Tafreshi et al., 2016), we had observed either no significant change (at α = 20°) or significant reduction of HR at tilt angles above α = 20° (Sarabadani Tafreshi et al., 2016) in response to PE alone, and a significant increase in response to head-up tilting alone (average 16 bpm at α = 60°). It is known that the baroreflex buffers fluctuations of BP that occur due to changes in position [e.g., head-up tilting (Schwartz and Stewart, 2012)], exercise (Raven et al., 2006) or other conditions. It can be depressed in older people (Jones et al., 2003) or patients with neurological disorder (Benarroch, 2008), leading to an increase of peripheral resistance and higher HR (Benarroch, 2008). Thus, we expected in our study population a baroreflex depression and consequently more pronounced changes in BP and HR. However, we only observed that in case of BP.

Furthermore, we looked at the effect of the change in PE frequency on the cardiovascular system. Only for sBP, we observed a trend toward higher values, but the effect was not clinically relevant (i.e., HR > 2.5 bpm and BP > 5 mmHg Sarabadani Tafreshi et al., 2017b) even when we doubled the PE frequency from 24 to 48 steps per min. This result is in contrast to the current belief in the clinical application of robot-assisted tilt tables that higher PE frequency would result in higher effects. We conclude that the relationship of cardiovascular variables and PE is not proportional, i.e., PE frequency does not scale to cardiovascular variables. Rather, it reaches a plateau where a higher increase of PE frequency would not result in any systematic cardiovascular changes. Still, PE (independent of its frequency), increases sBP and dBP and does so especially at high tilt angles. This effect might have the potential to compensate the drop in BP due to orthostatic challenge and, as a consequence, prevent syncope during head-up tilt. This is a consistent conclusion also supported by previous studies (Chi et al., 2008; Yoshida et al., 2013).

When combining PE with head-up tilting, PE had an additive effect on increasing both sBP and dBP. However, as the mean BP drop in response to head-up tilting in our study was not significant, we can neither conclude nor reject that PE could have a systematic compensatory effect to prevent sBP or dBP drop and potentially syncope.

The day effect was not significant in any of the analyses, indicating that the observed effects of PE on the cardiovascular variables are consistent from day to day. However, the result mirrors the average, and it cannot be concluded that PE always changes these variables in the same direction or same amount in a single subject.

We developed independent statistical models for relative changes of HR, sBP, and dBP, although these signals were recorded during the same experiments. We did not correct the reported *p*-values for multiplicity (Sare et al., 2009) because the cardiovascular variables are coupled and various mechanisms such as the baroreflex circuit (Jones et al., 2003; Mukkamala et al., 2003; Raven et al., 2006; Benarroch, 2008; Schwartz and Stewart, 2012) govern the relationship between them. However, the degree of this dependency during head-up tilt or PE is not clear to us. In particular, baroreflex resetting [e.g., during exercise (Raven et al., 2006) or orthostatic challenge (Schwartz and Stewart, 2012)] makes investigation of such dependencies more challenging. Therefore, although we acknowledge this limitation of our study, we avoided any systematic correction for the multiplicity of our reported *p*-values.

## 5. Conclusion and outlook

We provide evidence that PE can increase sBP and dBP of neurological patients during head-up tilt. However, in our population with mild BP changes, we could not conclude a preventive effect of PE on orthostatic hypotension and syncope. A study with a more homogenous patient population is necessary to underpin the preventive effect of PE on orthostatic hypotension and syncope in neurological patients. Furthermore, we observed that similar to healthy subjects, the effect of PE on sBP is dependent on the verticalization angle.

To our knowledge, this is the first study which systematically investigated the effect of PE frequency on the cardiovascular system's response. We found that modulation of the PE frequency does not play an important role in PE influence. Hence, PE frequency cannot be used as a reliable variable to modulate PE influence. Thus, investigation of other modalities such as foot loading to modulate and enhance the PE effect is a potential future work.

## Author contributions

AS, RR, and VK conceived the study. AS designed and coordinated the study, and collected and analyzed the data. AS, RR, and VK interpreted the data. AS wrote the first draft of the manuscript. AS, RR, and VK revised the manuscript.

### Conflict of interest statement

The authors declare that the research was conducted in the absence of any commercial or financial relationships that could be construed as a potential conflict of interest.

## Abbreviations

AIC: Akaike information criterion
BIC: Bayesian information criterion
BMI: Body mass index
BP: Blood pressure
bpm: Beats per min
CI: Confidence interval
CIP: Critical Illness Polyneuropathy
CVA: Cerebrovascular accident
dBP: Diastolic blood pressure
FES: Functional electrical stimulation
HIE: Hypoxic Ischemic Encephalopath
HR: Heart rate
LRT: Likelihood ratio test
ML: Maximum likelihood
PE: Passive robotic leg exercise
REML: Restricted maximum likelihood
sBP: Systolic blood pressure
SE: Standard error.
